# Radiologic evaluation of scrotal lesions: A case of polyorchidism with abnormal ultrasound findings

**DOI:** 10.1016/j.radcr.2025.09.056

**Published:** 2025-10-04

**Authors:** Aileen Zhang, Erin Gomez

**Affiliations:** a Johns Hopkins University School of Medicine, 1600 McElderry St, Baltimore, MD 21205, USA; b Johns Hopkins Medicine Department of Radiology and Radiological Science, 733 N Broadway, Baltimore, MD 21205, USA

**Keywords:** Supernumerary testis, Polyorchidism, Tubular ectasia, Spermatocele

## Abstract

Supernumerary testis (SNT), or polyorchidism, is a rare congenital condition characterized by the presence of 3 or more testes. Here, we present the case of a 53-year-old man with a history of prostate cancer who presented with a painless right-sided scrotal mass and abnormal ultrasound (US) findings. US imaging showed a heterogeneous and hyperechoic paratesticular mass, initially raising concern for a SNT, primary neoplasm, or metastatic process. Magnetic resonance imaging (MRI) was obtained for further evaluation of suspicious features and ultimately confirmed the diagnosis of SNT, allowing for conservative management. Given the imaging findings, the patient did not require surgical intervention and continued routine follow-up with annual testicular exams and prostate-specific antigen levels.

## Introduction

Supernumerary testis (SNT), or polyorchidism, is a rare congenital condition that involves the presence of 3 or more testes. Since the first histologically confirmed case of SNT in 1880, approximately 200 human cases of SNTs have been reported in literature [1,2]. SNTs tend to be left-sided and can be intrascrotal or ectopic in location, such as in the inguinal canal and abdominal cavity [3]. In addition to location, size, and quantity, SNTs differ in reproductive potential based on sperm quality, degree of active spermatogenesis, and presence of a draining epididymis and ductus deferens [3]. While patients with SNTs often present with a mobile and painless scrotal mass, an estimated one third of cases are diagnosed incidentally during the evaluation of other conditions such as inguinal hernia or testicular torsion [2]. Half of SNTs are diagnosed between ages 15 to 25, with a mean age of diagnosis at 17 [3,4]. Since polyorchidism has been associated with an increased risk of torsion, infertility, and malignancy, the timely identification of SNTs can facilitate early and regular monitoring or surgical intervention for patients [4]. Currently, ultrasonography (US) is the first-line modality for detecting SNTs. While US is a highly sensitive modality for the identification of paratesticular lesions, the lower specificity of US features can limit the differentiation between benign and malignant phenomena [5]. In cases with abnormal US findings, magnetic resonance imaging (MRI) can play a complementary role in the further characterization and diagnosis of paratesticular masses.

## Case report

A 53-year-old man with a past medical history of prostate cancer presented with a firm, painless right scrotal mass. He was otherwise asymptomatic, without fever or chills, weight changes, urinary symptoms, or skin changes. The patient first noticed and palpated the lesion on self-exam several years ago, but further evaluation was delayed when he was diagnosed with prostate cancer, requiring radical prostatectomy and lymph node dissection. Histopathology showed adenocarcinoma with extraprostatic extension, negative margins, and no metastasis to regional lymph nodes. His prostatectomy was complicated by erectile dysfunction, which was responsive to intracavernosal injections. The patient also had prediabetes, hypertension, and hyperlipidemia. He had no other significant surgical history. On physical exam, the patient was well-appearing and afebrile with appropriate vitals. Genitourinary exam showed bilaterally descended testes, no penile lesions or plaques, and a hard, mobile, marble-sized mass in the right hemiscrotum. He had no abnormal abdominal or neurologic findings. Since his radical prostatectomy, his annual prostate-specific antigen (PSA) levels were undetectable (<0.1 ng/mL). His complete blood count (CBC) was within normal limits, and his comprehensive metabolic profile (CMP) showed mildly elevated total calcium (10.5 mg/dL), which has been a stable finding for the past 4 years. Previous ionized calcium levels were within normal limits.

**Fig. 1:**
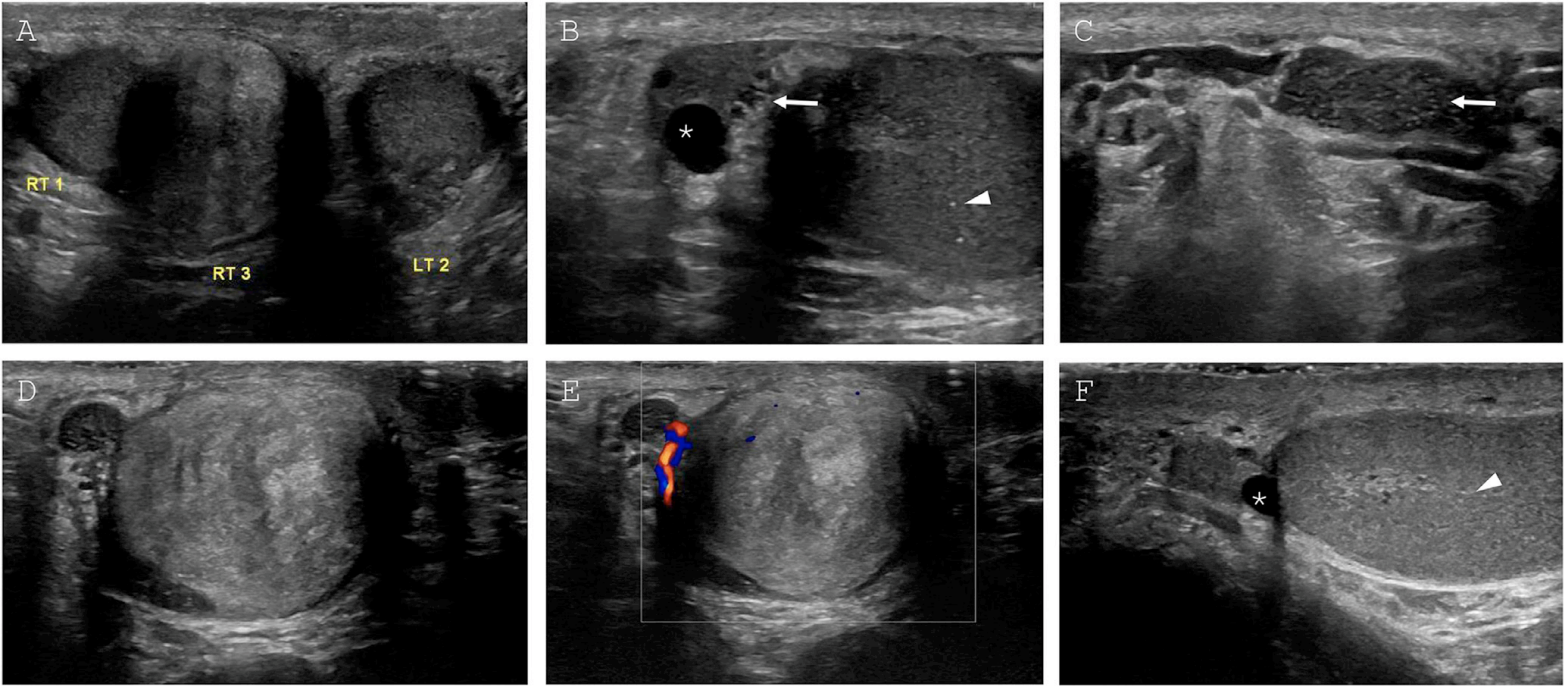
**(**A) Grayscale (A-D, F) and color (E) scrotal US images. Side-by side testicular view (A) demonstrating bilateral descended testes (RT 1, LT 2) with homogenous echotexture. Paratesticular mass (RT 3) is inferior, medial, and separate from right testis. (B) Sagittal view of right epididymal head with anechoic cyst or spermatocele (asterisk) and tubular ectasia (arrow). Right testis with scattered microlithiasis (arrowhead). (C) Sagittal view of right epididymal tail with tubular ectasia (arrow). (D and E) Transverse view of right paratesticular mass with heterogenous echotexture and minimal vascularity. (F) Sagittal view of left scrotum with anechoic epididymal cyst or spermatocele (asterisk) and left testis with scattered microlithiasis (arrowhead).

Scrotal ultrasonography was performed and confirmed descended testes bilaterally with a separate right-sided paratesticular mass (Fig. 1). There was no sonographic evidence of varicocele or testicular torsion bilaterally. The left testicle, which measured 3.4 × 1.7 × 3.0 cm, had homogenous echotexture with minimal microlithiasis. The right testis, which measured 3.5 × 2.0.x 3.0 cm, had homogenous echotexture with minimal microlithiasis. There was also a trace right-sided hydrocele, right epididymal head cyst, and tubular ectasia of the right epididymis. The right paratesticular mass, which measured 2.5 × 2.7 × 2.2 cm, was located inferior and medial to the right testicle and demonstrated internal color Doppler flow. Of note, the lesion was heterogenous and hyperechoic when compared to the normal testes. Since US findings confirmed the paratesticular nature of the right-sided scrotal mass, differential diagnoses favored benign (SNT, adenomatoid tumor) or malignant (adenocarcinoma, sarcoma) paratesticular lesions over a primary testicular neoplasm [6]. For this reason, measurement of testicular cancer markers like alpha-fetoprotein and β-human chorionic gonadotropin, classically produced by testicular germ cell tumors [7], were deferred at the time. Given the abnormal imaging features observed in the scrotal mass, metastasis and paratesticular malignancy could not be excluded and pelvic MRI was performed for further characterization.

**Fig. 2:**
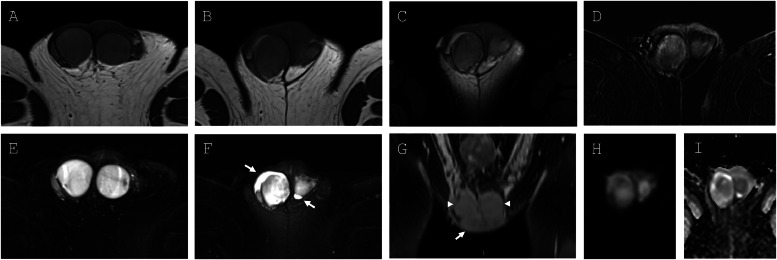
**(**A) T1-weighted axial images of the testes and (B) right SNT, demonstrating homogenous, intermediate signal intensity. (C) T1-weighted axial image of right SNT shows no signal dropout with fat saturation, confirming absence of intralesional fat. (D) T1-weighted postcontrast axial image of right SNT shows heterogeneous enhancement, suggestive of previous vascular or infectious insult. (E) T2-weighed axial fat saturated images of the testes and (F) right SNT, with similarly homogenous T2 hyperintensity. Bilateral hydroceles present (arrows). (G) T1-weighted postcontrast coronal image of bilateral testes (arrowheads) and right SNT (arrow). (H and I) Diffusion-weighted images and corresponding ADC map demonstrating low-level diffusion restriction within the right SNT.

**Fig. 3:**
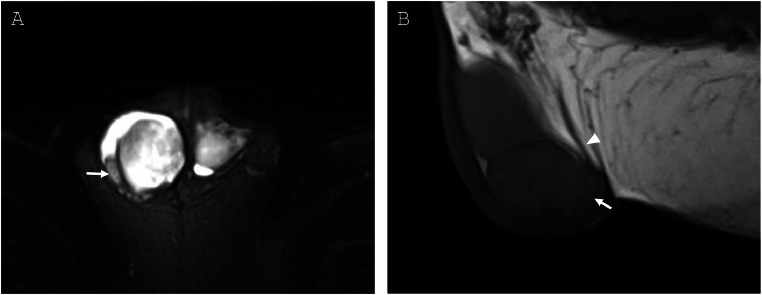
**(**A) T2 axial fat saturation image with draining epididymis (arrow) of right-sided supernumerary testicle. (B) T1 sagittal nonfat saturation image with draining epididymis (arrow) and ductus deferens (arrowhead) posterior to supernumerary testicle.

On MRI, the right paratesticular mass was T2 hyperintense and homogenously T1-isointense with heterogenous peripheral contrast enhancement (Fig. 2). There was no evidence of intralesional fat. Mild tissue heterogeneity and low-level diffusion restriction were observed within the lesion. Given the MR imaging features and similarity to the right and left testes, the lesion was identified as an intrascrotal supernumerary testicle with a draining epididymis and ductus deferens (Fig. 3). It was proposed that the heterogeneous peripheral enhancement of the SNT may have been secondary to prior infectious or vascular injury. In addition, MRI demonstrated trace bilateral hydroceles, left-sided varicocele, and small bilateral inguinal hernias. Given the MRI findings, the patient’s urologist advised that surgical intervention and additional follow-up imaging were not warranted. His primary care physician continued to monitor his annual PSA levels for biochemical recurrence of his prostate cancer, as well as for interval change in the supernumerary testis on physical exam.

## Discussion

Scrotal US is considered a first-line modality for identifying SNTs, with MRI often serving as an adjunct imaging technique. On US, SNTs appear as well-circumscribed ovoid structures that are homogeneously isoechoic or hypoechoic and have similar or reduced vascularity as compared to normal testes [8]. While US has almost 100% sensitivity for the identification of scrotal lesions, US has a lower specificity of 70% to 90% due to its limited tissue characterization, operator dependence, and limited field of view [5]. For this patient, the paratesticular mass was heterogeneous and hyperechoic on scrotal US, appearing dissimilar from the neighboring testes and raising concern for a primary paratesticular neoplasm or metastasis. Malignant tumors such as liposarcomas and leiomyosarcomas can appear on US as well-defined paratesticular masses with heterogenous areas of echogenicity [5]. Ultimately, differentiation between benign and malignant paratesticular masses on US imaging can be challenging, even with advanced sonographic techniques like strain elastography (SE). In a recent case report by Corvino and colleagues, a benign adenomatoid tumor mimicked the appearance of malignancy by presenting with heterogenous echogenicity, internal color Doppler flow, and elevated tissue stiffness on SE [9]. The patient subsequently underwent surgical excision of the benign paratesticular mass, an intervention that could have been avoided if initial US features were more specific. When scrotal US findings remain ambiguous, complementary imaging modalities can further evaluate lesions and guide clinical management. In this case, MRI was critical to differentiating between benign and malignant pathology.

On MRI, SNTs appear similarly to normal testes as homogenous ovoid structures with T2 hyperintensity, T1 iso- or hypointensity, diffusion restriction, and contrast enhancement [10]. This patient’s paratesticular mass exhibited a similar appearance to normal testes on T1- and T2-weighted images. Lack of signal attenuation on fat saturation sequences ruled out a lipoma or liposarcoma, and lack of cystic foci, thickened septa, tissue invasion, and abnormal contrast enhancement lowered suspicion of primary or secondary malignancy [10]. For this patient, MR imaging was highly suggestive of benign supernumerary testis.

**Fig. 4:**
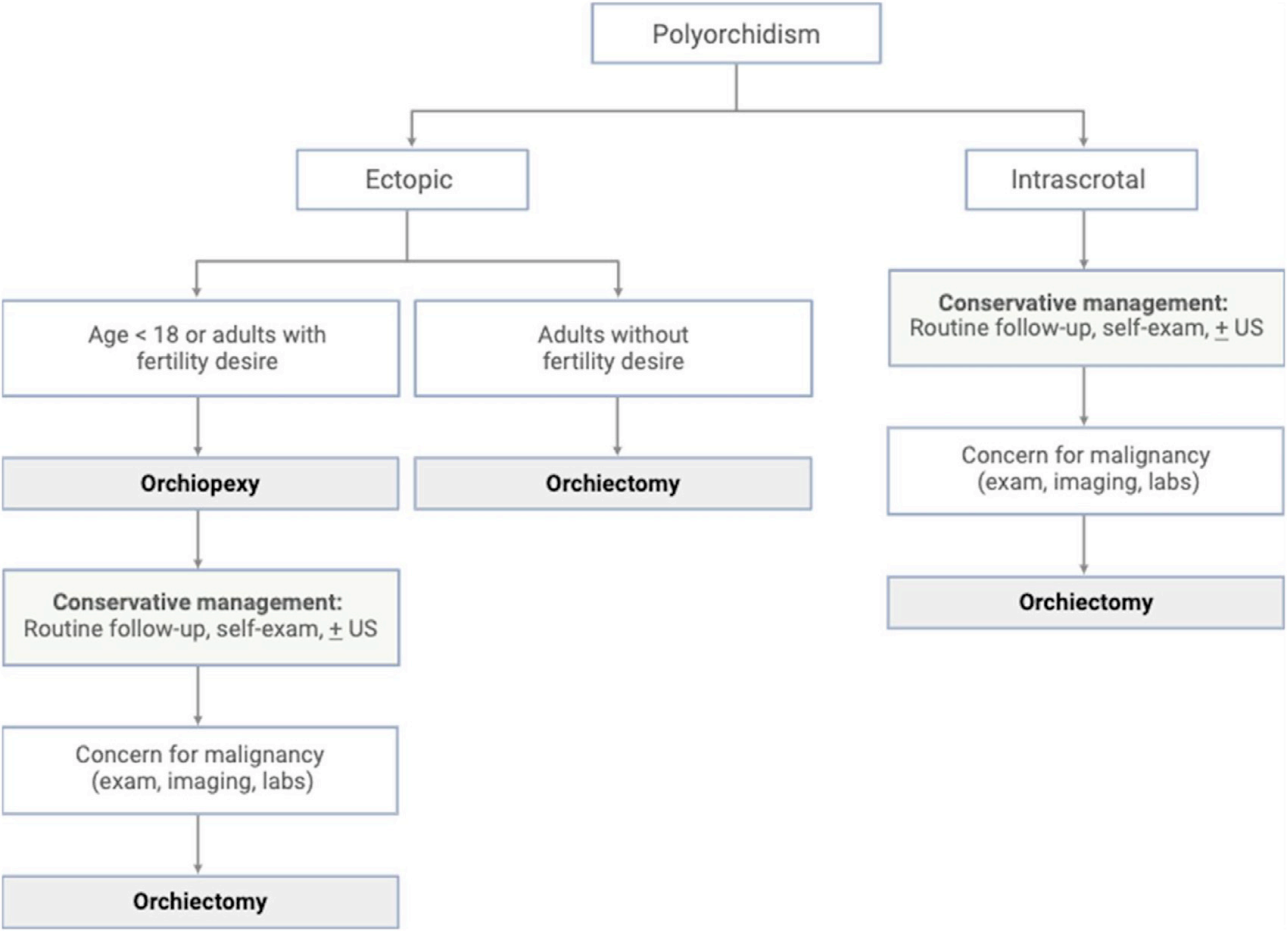
Schematic for proposed clinical management of supernumerary testes, adapted from “An Up-to-Date Systematic Review” by Balawender et al. [2] under Creative Commons Attribution 4.0 International License (CC BY 4.0).

The management of SNTs can involve orchiectomy, orchiopexy, or conservative monitoring with follow-up scrotal US as needed. While orchiectomy was historically favored due to concern for malignancy, advances in imaging have allowed for increasingly conservative and individualized management of SNTs [4]. The management approach towards SNTs often strikes balance between oncologic risk and fertility preservation. Polyorchidism has been associated with increased risk of malignancy, with 4% of reported SNT cases developing testicular malignancy [2]. The high prevalence of cryptorchid SNTs may contribute to the link between SNTs and testicular cancer. Cryptorchidism has been associated with a 3.7- to 7.5-fold increase in testicular cancer risk [11], and a quarter of SNTs have been found either in the inguinal canal or abdominal cavity [2]. Polyorchidism has also been associated with infertility [3]. Some authors suggest that orchiectomy may result in the unwarranted removal of functional testicular tissue [3,12], as 50-65% of SNTs have signs of active spermatogenesis [3] and 80% of SNTs are drained by an epididymis and ductus deferens [2]. Based on these considerations, recent recommendations regarding the management of SNTs account for location, reproductive potential, and presence of suspicious features. Patients with intrascrotal SNTs can undergo conservative management with regular self-exams and follow-up US imaging as needed (Fig. 4). Any signs of malignancy on imaging, physical exam, or laboratory evaluation would warrant orchiectomy [4]. Patients of reproductive age with ectopic SNTs can pursue orchiopexy with observation, while patients beyond reproductive age with ectopic SNTs should pursue orchiectomy [2]. In this patient case, MRI played an important role in guiding the management approach of the SNT. Since this was an intrascrotal SNT without suspicious features, the patient was recommended conservative management.

Other findings of note on the patient’s diagnostic imaging included cysts or spermatoceles and tubular ectasia of the bilateral epididymides. Spermatoceles are fluid-filled cysts that may contain sperm, while tubular ectasia refers to the cystic dilation of tubules. Both spermatoceles and tubular ectasia are known sequelae in patients following vasectomy [13]. To our knowledge, this is the first reported case of epididymal tubular ectasia following radical prostatectomy, rather than postvasectomy. Since tubular ectasia and cysts develop due to high intraluminal pressures from distal obstruction of the ductus deferens following vasectomy, it is understandable that both phenomena could also develop following ligation of bilateral ductus deferens during radical prostatectomy. Asymptomatic tubular ectasia and spermatoceles do not warrant treatment, but both phenomena can be associated with chronic scrotal pain [14]. Although additional studies are needed, this novel case of tubular ectasia may help contextualize epididymal changes and chronic scrotal pain in patients following radical prostatectomy.

In summary, we present the case of a 53-year-old man with a history of prostate cancer with a mobile, painless right-sided scrotal mass. Initial ultrasound evaluation demonstrated features indeterminate but suspicious for malignancy. This prompted an MRI of the pelvis, which helped confirm the diagnosis of supernumerary testicle without malignant features, which ultimately guided conservative management.

## Patient consent

The patient reported in the manuscript signed the informed consent/authorization for participation in research which includes the permission to use data collected in future research projects, including the presented case details and images used in this manuscript.
